# Absence of HPV Infection Is Associated with Smoker Patients with Squamous Cell Carcinoma of the Oropharynx

**DOI:** 10.1155/2014/371570

**Published:** 2014-09-30

**Authors:** Gláucia Resende Soares, Adriana Demathé, Neivio José Mattar, Éder Ricardo Biasoli, Glauco Issamu Miyahara

**Affiliations:** ^1^Oral Oncology Center, Department of Pathology and Clinical Propedeutics, Araçatuba School of Dentistry, Universidade Estadual Paulista (UNESP), Brazil; ^2^Araçatuba Pathology Institute, Brazil; ^3^Oral Oncology Center, Department of Pathology and Clinical Propedeutics, 1193 José Bonifácio Street, 16015-050 Araçatuba, SP, Brazil

## Abstract

The purpose of this study was to evaluate the survival of patients with SCC of the oropharynx, according to the presence of HPV and tobacco consumption. A total of 37 patients were followed up for at least 5 years after being diagnosed with SCC of the oropharynx. The biopsy tissue was submitted to the polymerase chain reaction (PCR) and in situ hybridization (ISH) methods for broad determination of HPV presence, to identify the presence of high-risk viruses (16 and 18). 12 of the 37 (32.4%) samples were HPV positive, whereas the two specific types of virus were identified in two samples for HPV-16 and in no samples for HPV-18. We observed no significant effect of the virus in survival analysis, irrespective of tobacco consumption. The level of tobacco consumption was significantly higher in the group of HPV-negative patients (*P* = 0.0283), in which all the patients in this group were smokers. Therefore, HPV did not change the survival of patients with SCC of the oropharynx in this study, indicating that factors other than tobacco need to be studied in conjunction with it, and the level of tobacco consumption is significantly higher in the group of HPV-negative patients.

## 1. Introduction

Considering that the etiological factors most correlated with the development of squamous cell carcinoma (SCC) of the head and neck are tobacco and alcohol consumption, this study evaluated the isolated and associated effect of these two carcinogens, showing that alcohol does not act in an isolated manner, as does tobacco, but the interaction of these habits presents a significant effect on the formation of the tumors [1]. When patients do not smoke and do not drink, infection by high-risk HPV is the predominant etiological factor for the development of SCC of the head and heck, which corresponds to around 10–20% of the cases [2, 3].

Smoking and infection by HPV are the main independent prognostic factors for patients with SCC of the oropharynx, probably because they determine the molecular profile of cancer and, therefore, the response to therapy [4, 5]; however, Laco et al. [6] affirmed that smoking in an isolated manner does not appear to be an important prognostic factor.

The smoking habit is very common in patients with SCC of the head and neck, ranging from 66.6% to 89.4 in the studied population [1, 7, 8]. The prevalence of HPV in these patients shows considerable differences from one study to another, whereas HPV has been found in 32% [9], 63.8% [4], and 81.3% [10] of the cases. In addition to these data, we have available two review studies that evaluated the prevalence of HPV related by over thirty articles each one, with a mean value of 41% of HPV-positive patients being found [7] and in a later study the mean value of 51% [11]. When viral typing was performed, the articles indicated that HPV type 16 was the most prevalent [4, 7, 9–11].

The correlation between tobacco consumption and the presence of HPV in patients with SCC of the oropharynx indicated that the number of smokers is higher in the group of HPV-negative patients and that after the science of diagnosis 66% of the smoker patients continued with the habit, in contrast with HPV-positive patients. This last one presented a lower level of tobacco consumption, and only 30% of the smoker patients continued with the habit after the science of diagnosis [8], as in the study of Mizumachi et al. [9], who verified a significantly higher level of tobacco consumption in the group of HPV-negative patients (*P* = 0.0003).

As regards the influence of these two above-mentioned etiological factors, tobacco and infection by HPV in the local recurrence of SCC of the oropharynx, the study indicated that the group of HPV-positive patients did not undergo changes in the recurrence of the lesion, according to the consumption of tobacco, or not [8]. However, the group of HPV-negative smoker patients who continued with the habit after the science of diagnosis presented a higher rate of local recurrence of the lesion when compared with those HPV-negative smoker patients who quit the habit after the diagnosis [8].

With regard to the risk for developing SCC of the head and neck in individuals with HPV-16, in spite of Dayyani et al. [7] having evaluated that the risk is greater in this population, treatment has been shown to be more effective in this group of patients, showing lower levels of recurrence and progression of the disease, in addition to presenting better overall survival [2, 7, 11–13]. We believe that there are many uncertainties with respect to the effect of tobacco consumption and infection by HPV on the survival of patients with SCC of the oropharynx. Thus, the purpose of this study was to evaluate the survival of patients with squamous cell carcinoma of the oropharynx, according to the presence of HPV and tobacco consumption.

## 2. Materials and Methods

### 2.1. Study Population

The study group was composed of 37 patients with carcinoma of the oropharynx, treated with curative intention at the Oral Oncology Center, UNESP, between 2005 and 2007. The study was approved by the Ethics Committee on Research in Human Beings, of the Araçatuba School of Dentistry (Protocol 2005/00689). The data were obtained from the patients' record chards, and follow-up was performed for at least five years.

All cases underwent identical personal interviews regarding gender, age, tobacco smoking, and alcohol drinking. Patients reporting having never consumed at least one cigarette or one drink at a regular monthly basis were considered nonusers. Cumulative doses of tobacco exposure were calculated in terms of pack-years (one pack-year equals to one package of cigarettes smoked daily for one year) [1]. Most patients (25/37) presented with advanced clinical stage at diagnosis, and then the majority of patients (31/37) received a combination of chemotherapy and radiotherapy as treatment choice.

### 2.2. Laboratory Studies

The paraffinized tissue samples were submitted to the DNA extraction technique (QIAamp DNA Mini Kit, QIAGEN Ltd., Crawley, UK), DNA purity, and quantification test (NanoDrop ND-1000 UV-Vis) and PCR for gene control of human *β*-globin (PC03 and PC04, Life Technologies). A cancer sample was considered HPV-positive after the nPCR method had been performed in triplicate, with the use of the following primers: GP5+ and GP6+ (Life Technologies).

Histological cuts of 5 *μ*m were placed on silanized slides (SIGMA) and submitted to the in situ hybridization reaction (ISH), using specific biotinylated probes for HPV 16 and 18 (DAKO). The ISH reactions were accompanied by a positive control (Kit GenPoint-DAKO), negative control (kit GenPoint-DAKO, subtracting the probe), and reaction control, using a normal oral mucosa biopsy.

### 2.3. Statistical Analysis

Comparisons were made between the clinical characteristics of the HPV-positive and HPV-negative cancers, using the Exact Fisher or chi-square test, with statistical significance being established at *P* < 0.05. The overall survival curves were calculated by the Kaplan-Meier method. Survival was calculated from data at the beginning of diagnosis until death or the last date at which the patient was known to be alive. Statistical significance of the differences between the survival time was determined by the log rank test at a level of significance of *P* < 0.05.

## 3. Results

In total, 12 of the 37 (32.4%) tumor tissue samples were shown to be HPV-positive. By means of investigating the two specific types of HPV (16 and 18), two samples (16.6%) were positive for HPV-16 and no sample was positive for HPV-18.

Most patients were diagnosed with 45 years of age or older (70.3%), and there was no statistically significance difference between the age of the patients and the HPV status (*P* = 0.4870). The male sex was predominant in our study group (89.1%) and 75% of the samples positive for HPV were from patients of the male sex (9/12), with the *P* value = 0.0908 (Table 1).

As regards the ingestion of alcoholic beverages, we observed that the samples of 66.6% of the consumers were not positive for HPV; however, among the abstainers 71.4% also formed part of the HPV-negative samples, leading to *P* = 0.5937. The level of tobacco consumption was high in the group evaluated (91.9%), but this habit was significantly higher in the HPV-negative than in HPV-positive patients (*P* = 0.0283). The advanced clinical stage was observed in 67.6% of patients at the time of diagnosis (25/37), and the proportion of HPV-positive patients was higher in advanced stage oropharyngeal cancer (2,78 : 1), when compared to early stage oropharyngeal cancer (4 : 1), however not statistically significant (*P* = 0.6092, Table 1).

**Figure 1:**
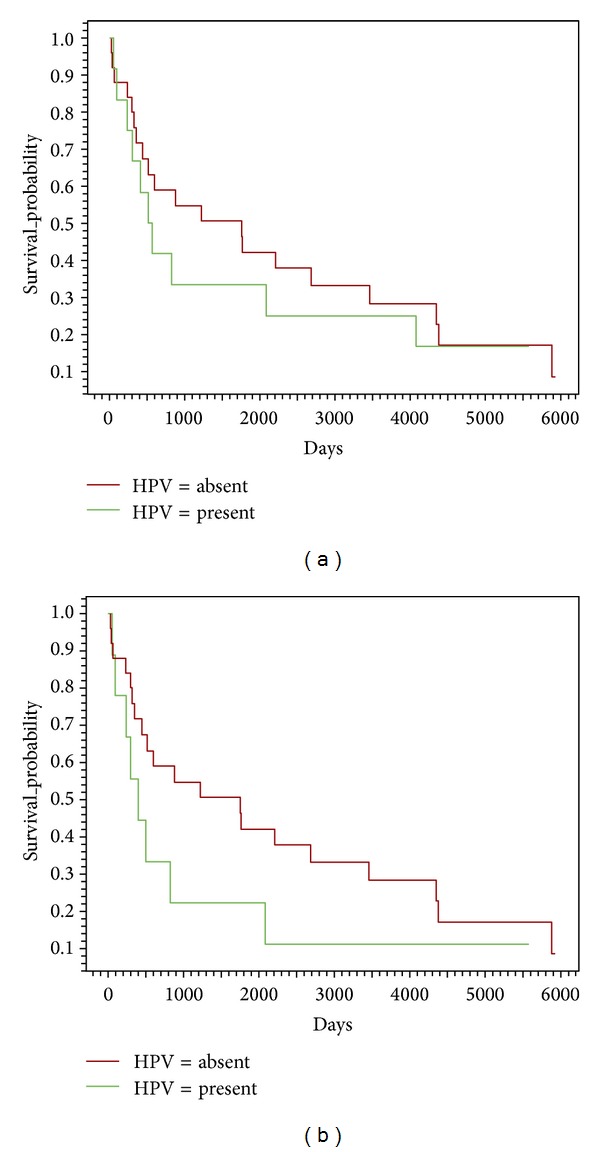
Survival analysis of patients with and without the presence of virus, by the Kaplan-Meier method, and by means of the Log-Rank test Epi Info program version 3.5.4 (July 30, 2012). Analyses were performed on the whole cohort ((a); *N* = 37) and on smoker patients ((b); *N* = 34). Differences were considered significant when *P* < 0.05.

The studied patients, in general, had a low survival rate; however, in the group of HPV-positive patients, only 33% remained alive after five years from the time of diagnosis, a datum similar to that found for the group of HPV-negative patients (40%). The Log-Rank test in accordance with viral presence and absence was applied in two ways; in the first test, all the patients were included irrespective of tobacco consumption (*P* = 0.5416; Figure 1(a)), whereas in the second test, only smoker patients were evaluated, with *P* value = 0.1773 (Figure 1(b)).

## 4. Discussion

The HPV-positive patients with SCC of the head and neck, who presented a better survival, were frequently of the male sex with low alcohol and tobacco consumption [11]; in our study, 75% of the HPV-positive patients were of the male sex; however, the majority were alcohol and tobacco consumers (89%) and presented no similarity to the above-mentioned study with regard to alcohol and tobacco consumption, nevertheless corroborating the classical profile of patients with SCC [6, 14].

The prevalence of HPV in patients with SCC of the oropharynx ranges between 32 and 81% [4, 9–11] and their distribution may differ according to the continent in which the study is conducted, with a prevalence of 47% being related in North America, 46% in Asia, 36% in South/Central America, Australia, and Africa, and 26% in Europe [15]. Our study identified the presence of virus in 32.4% of the samples of patients with SCC of the oropharynx, a datum similar to that related by Dayyani et al. [7], in addition to being compatible with the viral prevalence data for South America, according to the study of Kreimer et al. [15]. However, when we evaluated the type of HPV most associated with these lesions (HPV-16), we verified that only 17% of the samples presented the virus, indicating low prevalence of HPV-16.

The patients with SCC of the oropharynx presented an overall survival that changed according to the presence of HPV. Studies have pointed out that there is an improvement in the survival rate in five years of HPV-positive patients (71–80%), when compared with HPV-negative patients (36–50%) [4, 16, 17]; however, this correlation of virus with an improvement in survival was not observed in our study. Considering that our group presented a high prevalence of smokers (91.9%) and low prevalence of HPV-16 (17%), we believe that these data changed the survival of patients, because of being independent prognostic factors, as has been elucidated in previous studies [4, 5].

In addition to various studies having affirmed an improvement in the prognosis of SCC of the oropharynx due to the presence of HPV [2, 4, 9, 16], these patients were less exposed to tobacco and alcohol, which also contributed to a better prognosis, and when the Kaplan-Meier statistical test was applied, statistically significant data were observed [9, 18], whereas, in our study, the statistical analysis demonstrated that the presence of HPV did not change the patient survival results.

In spite of the survival of smoker patients with SCC of the oropharynx not being benefited by the presence of HPV, as much as the nonsmoker patients were, the authors showed that the HPV-positive smoker patients presented better survival when compared with the HPV-negative smoker patients [4, 8, 19]. In this study, we observed no statistically significant difference between the survival of HPV-positive smoker patients and HPV-negative smoker patients (*P* = 0.1773).

When evaluating tobacco consumption by HPV-negative patients, studies have shown that this habit is significantly more prevalent in the group of HPV-negative patients [8, 9] as was found in this study in which all the HPV-negative patients were smokers, presenting a statistically significant value (*P* = 0.0283). Certainly, other factors, such as the expression of p16 of the epidermal growth factor receptor (EGFR) and the association of single nucleotide polymorphisms (SNPs), may contribute to the survival results [20, 21].

## 5. Conclusion

According to our study group, sex and alcohol consumption did not present correlation with the presence of the virus; presence of HPV does not indicate a significant improvement in the survival of patients with SCC of the oropharynx, and the level of tobacco consumption is significantly higher in the group of HPV-negative patients.

## Figures and Tables

**Table 1:** Comparisons between the clinical characteristics of the HPV-positive and HPV-negative cancers of the oropharynx, using the Exact Fisher or chi-square test.

Variables	Patients (*n* = 37)	HPV+ (*n* = 12)	HPV− (*n* = 25)	*P* ^a^
Age				0.4870
31–44 years	11	4	7	
≥45 years	26	8	18	
Sex				0.0908
Male	33	9	24	
Female	4	3	1	
Alcohol				0.5937
Alcoholic	30	10	20	
Abstainer	7	2	5	
Tobacco				0.0283∗
Nonsmoker	3	3	0	
Level-1 smoker^b^	14	3	11	
Level-2 smoker^b^	20	6	14	
Stages				0.6092
I or II	12	3	9	
III or IV	25	9	16	

^a^Exact Fisher or chi-square test. ^b^Cumulative consumption: level-1 smoker? 28 pack-years; level-2 smoker >28 pack-years. ∗Statistically significant value.
